# Cicada nymphs dominate American black bear diet in a desert riparian area

**DOI:** 10.1002/ece3.8577

**Published:** 2022-03-01

**Authors:** Erick J. Lundgren, Karla T. Moeller, Michael Otis Clyne, Owen S. Middleton, Sean M. Mahoney, Christina L. Kwapich

**Affiliations:** ^1^ Department of Biology Center for Biodiversity Dynamics in a Changing World (BIOCHANGE) Aarhus University Aarhus Denmark; ^2^ Section for Ecoinformatics and Biodiversity Department of Biology Aarhus University Aarhus C Denmark; ^3^ 7864 School of Life Sciences Arizona State University Tempe Arizona USA; ^4^ Ecology, Evolution, and Conservation Biology Graduate Program University of Nevada, Reno Reno Nevada USA; ^5^ 1948 School of Life Sciences University of Sussex Brighton UK; ^6^ Department of Wildlife Humboldt State University Arcata California USA; ^7^ 14710 Department of Biological Sciences University of Massachusetts Lowell Lowell Massachusetts USA

**Keywords:** American black bear, carnivore, diet, novel habitat, riparian

## Abstract

American black bears are considered dependent on high‐elevation forests or other montane habitats in the drylands of western North America. Black bear sign, including that of cubs, was observed throughout the summers of 2015, 2016, and 2018 along a perennial desert river in the Sonoran Desert of Arizona. We analyzed the contents of 21 black bear scats, collected from May to October of 2016 and 2018. Apache cicada nymphs (*Diceroprocta apache*) were the dominant food item, occurring in 90% of scats and comprising an average of 59% of scat contents. In the process of excavating these nymphs, bears created large areas of turned‐over soil, a form of ecosystem engineering with potential implications for soils, vegetation, and fluvial geomorphology. Given that species distributions are shaped by physiological and ecological contexts, as well as anthropogenic legacies, it is possible that black bears once occurred more commonly in desert riparian systems prior to widespread agricultural development, hunting, and dewatering. Although more research is necessary, we suggest that desert riparian systems may be an alternative habitat for black bears. Better understanding the diet and habitat breadth of American black bears is important in the context of increasing landscape fragmentation and militarization in the U.S.‐Mexican borderlands.

## Graphical abstract

American black bears are generally considered forest habitat dependent species. However, we report on black bears in a riparian system in the Sonoran Desert, with an unusual diet dominated by cicada nymphs. Our results suggest that much remains unknown about the ecologies of even a well‐studied species like American black bears.

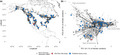

## 1 INTRODUCTION

Our understandings of organisms can be susceptible to research bias and historic or prehistoric range contractions (Britnell et al., 2021; Faurby & Svenning, 2015; Hughes et al., 2021). It is becoming increasingly clear that the habitat associations of some species reflect refugia from human persecution, instead of intrinsic biological or ecological requirements (Silliman et al., 2018), highlighting the need to document the ecology of apparently novel populations. Here, we report on the presence and diet of American black bears (*Ursus americanus*) occurring along the Gila River in the Sonoran Desert of Arizona.

American black bears are considered forest habitat specialists. In the Southwestern United States and Northwestern Mexico, forests are primarily restricted to higher elevation, montane habitats, which experience cooler temperatures and higher precipitation relative to lowland deserts (Delfín‐Alfonso et al., 2012; LeCount, 1980; Monroy‐Vilchis et al., 2016). Alternative forested habitats in this region are provided by riparian systems, where abundant water enables distinct forested habitats even in hyper‐arid desert landscapes. However, deserts are generally considered unsuitable bear habitat (Costello et al., 2001) and black bears are thought to only venture through lower elevation deserts when dispersing (Onorato et al., 2004), with little recorded about their utilization of desert riparian systems. American black bears have lost as much as 80% of their range in Mexico and were historically extirpated from large parts of the Southwestern United States (Monroy‐Vilchis et al., 2016), suggesting that these species may have once occurred more frequently in other habitat types (Lackey et al., 2013). Likewise, habitat loss from widespread alterations of riparian systems from historic and current dewatering, damming, and conversion to agriculture has led to many dramatic changes in these ecosystems and potentially the extirpation of many of their historic animal constituents (Leopold, 1970).

Over three summers, we continuously observed black bears and their sign, including dependent cubs and adults, within the riparian forests and floodplains of a perennial river in Arizona Upland Sonoran Desertscrub (Brown, 1994), a habitat type previously classified as “strongly avoided” by black bears (Atwood et al., 2011). Documenting the ecology and presence of species in this unique habitat can provide insight into the potential past and future distribution of black bears in North America and help us understand the potential ecologies of this species.

We report on the diet of these desert black bears, which we assessed from 21 scats collected over two summers. We then quantified the ecological distinctiveness of this habitat for bears by comparing it to all other reports of American black bear occurrences and all published diet studies.

## 2 METHODS

**FIGURE 1:**
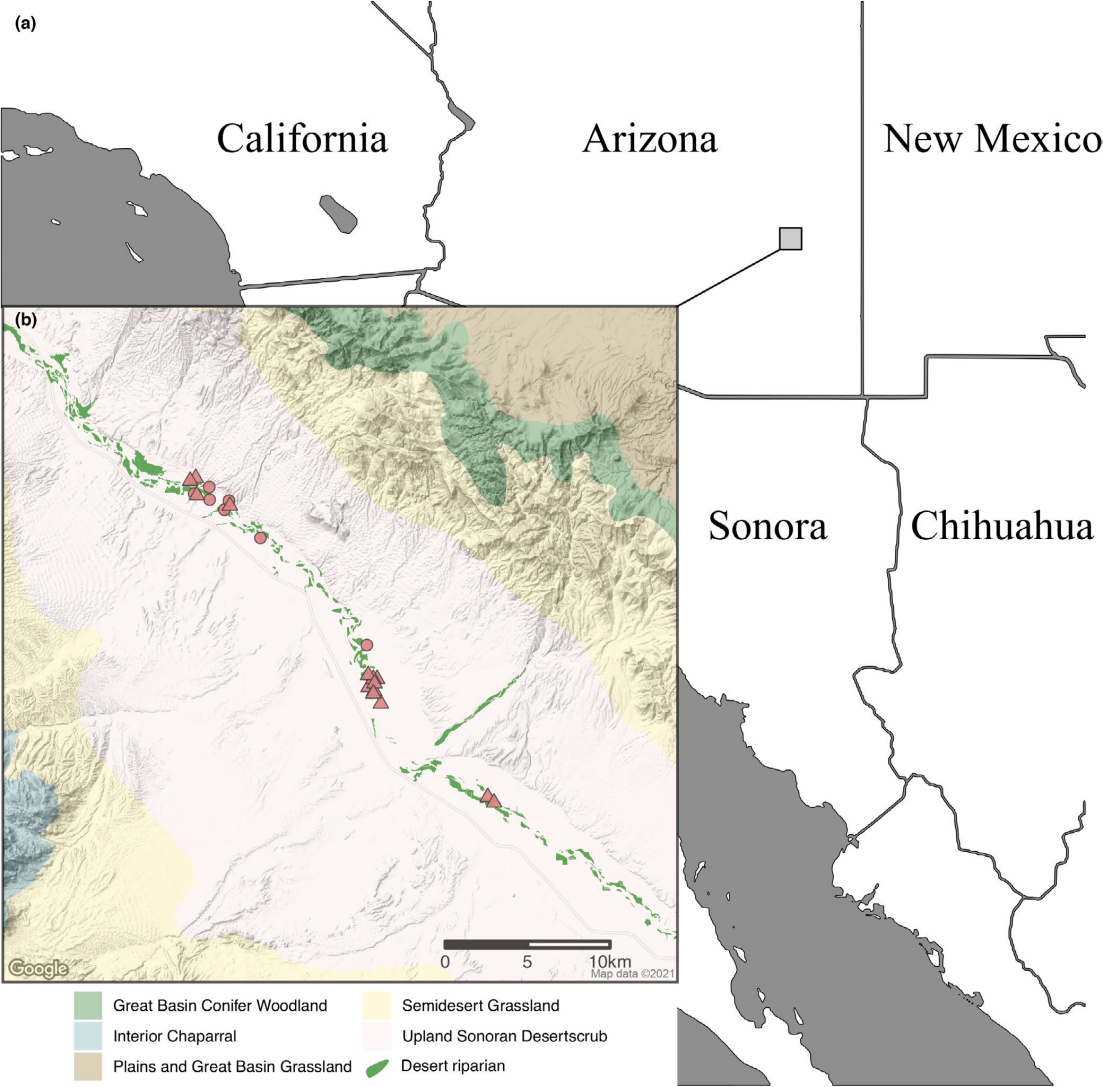
Location of Gila River black bears and local habitat types. (a) Southwestern United States and Northwestern Mexico. Gray square indicates location of Gila River. (b) Scat sample locations in 2016 (circles) and 2018 (triangles) along Gila River. Fill color of habitat indicates biotic community, following Brown (1994). Desert riparian habitat indicated in bright green polygons from U.S. Fish and Wildlife Service (2021)

Black bear scats were collected from May to October in 2016 and 2018 along a 20‐km section of the Gila River in Arizona, USA, at ca. 865 m above sea level (Figure 1). Upland vegetation adjacent to the riparian forests of the study site consisted of creosote (*Larrea tridentata*), mesquite (*Prosopis juliflora*), and ocotillo (*Fouquieria splendens*). A single bear scat was collected in this vegetation association, along an ephemeral tributary of the Gila River. The riparian communities of the Gila River, where the remaining bear scats were collected, were composed of Fremont cottonwood (*Populus fremontii*), Goodding's willow (*Salix gooddingii*), mule fat (*Baccharis salicifolia*), tamarisk (*Tamarix* spp.), and coyote willow (*Salix exigua*). Bears were also observed on higher floodplain terraces, which were forested by tamarisk and mesquite.

We visited sites approximately every 3 days while conducting southwestern willow flycatcher (*Empidonax traillii extimus*) surveys. We collected black bear scats opportunistically during surveys. Overall, we collected 21 scats, which we washed through a fine sieve to remove rocks, gravel, and the unidentifiable proteinaceous matrix. Individual dietary items were identified to lowest taxonomic order using taxonomic keys and field guides for arthropods and reference specimens at the Arizona State University Herbarium for fruit capsules and seeds. We did not identify herbaceous matter because the majority was sterile (i.e., no inflorescence) grass (but most likely Bermuda grass, *Cynodon dactylon*) or consisted of skeletonized and decomposed cottonwood leaves that might have been incidentally consumed.

We did not quantify bear population size at our sites; however, we frequently observed different‐sized bears and tracks, including those of cubs (e.g., approximately half the size of and closely associated with adult bear tracks). Because we collected samples on frequently visited trails, scats were likely to be less than one week old, but nonetheless we could not conclude if patterns in scat composition corresponded to seasonal shifts in diet as most scats were dry when collected. On average, scats were collected 10.5 days apart from each other (SD = 19.3, min = 0, max = 80), from May to October. While fresh bear sign was observed from May to August, we cannot verify that bears were full‐time residents of the Gila River.

We used two approaches to identify diet items in bear feces. For the first 7 samples (collected in 2016), after washing, we tore apart the fecal sample using fine forceps to isolate diet items. We then air‐dried sorted samples and measured their mass, which we divided by the total mass of the cleaned scat to calculate scat composition. Given the time‐consuming nature of this method, for the next 14 samples (collected in 2018), we employed a point‐intercept method, identifying the dietary item contacted by a grid of 50 vertical 1‐mm‐diameter pins (Ciucci et al., 2004). This method has been validated in comparison with dry‐mass methods for other species (Ciucci et al., 2004).

We calculated scat composition either as the mass of the dietary item divided by the scat's total dry mass or as the percentage of “contacts” per dietary item, for which we report mean and standard deviation across all scats for 2016, 2018, and combined. We further report the frequency of occurrence of each dietary item across all scats (e.g., the percent of scats containing any amount of each dietary item). This was based on the presence or absence of a dietary item, regardless of the dietary item's contribution to total scat composition.

To quantify the ecological distinctiveness of the Gila River black bears, we extracted georeferenced GBIF records (GBIF: The Global Biodiversity Information Facility, 2017) for American black bears from across their distribution. We included all occurrences with known methods and known dates to avoid spurious records. To avoid overemphasizing well‐sampled regions (e.g., Northeastern USA), we used the function “thin” in the R package “spThin” to reduce the number of occurrences per km^2^ (Aiello‐Lammens et al., 2015). Then, to understand the extent to which published literature on black bear diets is representative of their environmental distribution, we georeferenced all primary studies on black bear diets (*n* = 46), which we extracted from Web of Science using search terms “black bear,” “diet*,” “food,” and “*Ursus americanus*.”

**FIGURE 2:**
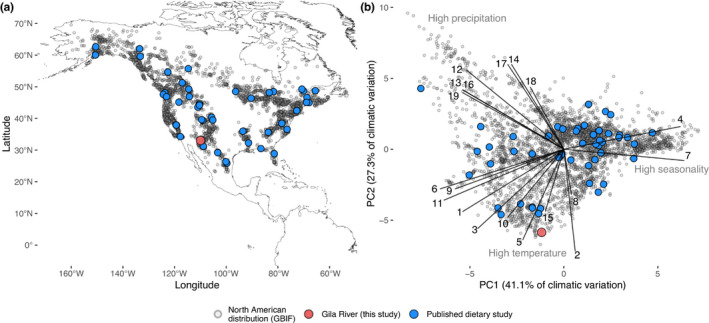
American black bear distribution across North America and across bioclimatic conditions. Small gray points indicate Global Biodiversity Information Facility (GBIF) occurrence records, red point indicates the Gila River bears, and blue points indicate published dietary studies (*n* = 44). (a) American black bear distribution in North America. (b) Dimensions of bear niche space are differentiated primarily (PC1) by seasonality and temperature, and to a lesser extent by precipitation (PC2). See Table A1 for PCA loadings. Bioclimatic variables are as follows: 1 = Annual °C, 2 = Diurnal °C range, 3 = Isothermality, 4 = °C seasonality, 5 = Max °C warmest month, 6 = Min °C coldest month, 7 = Temp. annual range, 8 = °C wettest quarter, 9 = °C driest quarter, 10 = °C warmest quarter, 11 = °C coldest quarter, 12 = Annual precip., 13 = Precip. wettest month, 14 = Precip. driest month, 15 = Precip. seasonality, 16 = Precip. wettest quarter, 17 = Precip. driest quarter, 18 = Precip. warmest quarter, 19 = Precip. coldest quarter

We then extracted 19 bioclimatic variables from WorldClim (Fick & Hijmans, 2017) for each black bear occurrence, the Gila River study site, and each published dietary study using the R package “raster” at a conservative 5 arc‐minute resolution (~71 km^2^ at study location) (Hijmans & van Etten, 2012). These variables encompass major drivers of vegetation and thermal tolerances, such as precipitation, temperature, and seasonal variability. We conducted a Principal Components Analysis (PCA) to synthesize these variables. We selected axis 1 and axis 2 as the realized niche space of black bears, which together explained 68.8% of total bioclimatic variability across bear occurrence records and dietary studies (Table A1). PC1 was negatively related to colder temperatures and positively related to seasonality and annual temperature range (Table A1, Figure 2). PC2 was negatively correlated with diurnal temperature range and maximum temperature, and positively correlated with precipitation (Table A1). Therefore, low PC1 and PC2 values represent areas that are consistently warmer and drier throughout the year (i.e., deserts), while higher PC1 and PC2 values are areas that are cooler, more mesic, and experience larger seasonal temperature shifts (i.e., temperate and montane systems, Figure 2).

## 3 RESULTS

Cicada nymphs were the most frequently encountered dietary item in bear scats, occurring in 90% of samples (19 of 21 scats). Cicada nymphs also constituted the majority of scat contents, with an average composition of 59% across all scats (SD = 36.6%, 2016 and 2018 data combined, Table 1). Nymph contribution to scat composition did not appear to vary seasonally: Samples in May had an average composition of 82.5%, while cicadas composed 99.9% of contents in a scat collected in October. Cicada nymphs were determined to be Apache cicadas (*Diceroprocta apache*) based on morphological characters, habitat affiliation, and geographic range (Sanborn & Phillips, 2013). Among other distinguishing characteristics, the observed nymphs lacked dark banding on the abdomen, and co‐occurring adults had a diagnostic white collar (Davis, 1921). *D*. *apache* is common along riparian terraces, often in association with Fremont cottonwood. However, *D*. *apache* can be easily confused with the more upland‐affiliated *D. semicincta*, which has been collected in the area (collected by Cazier and Gertsch in 1954; Gries et al., 2014). Recovered cicada nymphs had large wing buds that extended past the first abdominal tergites, indicating that they were in the 4th or 5th instar when ingested.

**TABLE 1:** Frequency of occurrence and scat composition of dietary items from 21 scats collected from the Gila River black bears

Item	Frequency of occurrence (%, number scats) across 21 scats	Scat composition (%)^a^ 2016 (mean ± SD)^a^	Scat composition (%)^b^ 2018 (mean ± SD)^b^	Scat composition, years combined (mean ± SD)
Apache cicada nymph (*Diceroprocta apache*)	90% (*n* = 19 scats)	74.2% (36.5)	51.4% (35.4)	59.0% (36.6)
Herbaceous vegetation	86% (18)	19.4% (26.0)	34.3% (31.2)	29.3% (29.8)
European honey bee (*Apis mellifera*)	33% (7)	1.4% (2.4)	3.4% (10.6)	2.7% (8.73)
*Ziziphus obtusifolia* seed	14% (3)	0.2% (0.5)	7.4% (18.9)	5.0% (15.7)
*Prosopis juliflora* seedpod	9.5% (2)	3.9% (10.3)	0.9% (3.2)	1.9% (6.4)
Tenebrionidae	5% (1)	0.002% (0.006)	0% (0)	0.0007% (0.003)
*Astragalus* seedpod	5% (1)	0.01% (0.03)	0% (0)	0.004% (0.02)
Rock squirrel (*Otospermophilus variegatus*)	5% (1)	1.0% (2.7)	0% (0)	0.3% (1.6)
Fish bones	5% (1)	0.0% (0)	0.3% (1.1)	0.2% (0.87)
Hair	5% (1)	0.0% (0)	0.9% (3.2)	0.6% (2.6)
Wood	5% (1)	0.0% (0)	0.9% (3.2)	0.6% (2.6)

Note

Frequency of occurrence of dietary items (%) across all scats was calculated from the number of scats containing each dietary item (at any quantity) divided by total number of scats. This included all scats collected in both 2016 (*n* = 7) and 2018 (*n* = 14). Scat composition in 2016 (*n* = 7) was calculated by measuring the mass of each dietary item extracted from the scat and dividing it by total scat mass. Scat composition in 2018 (*n* = 14) was calculated using the point‐intercept method. These results are reported separately and combined (*n* = 21).

^a^ Dry‐mass method used in 2016.

^b^ Point intercept method used in 2018.

Additional dietary items consisted of herbaceous vegetation (primarily *Cynodon dactylon*), found in 86% of scats, and comprising 29.3 of all scat contents (SD = 29.8). The European honey bee (*Apis mellifera*) was found in 33% of scats (2.7% of all scat contents, SD = 8.73). Other foods included graythorn seeds (*Ziziphus obtusifolia*) in 14% of scats (5.0% of scat contents, SD = 15.7) and mesquite seedpods (*Prosopis juliflora*), which were found in 9.5% of scats (mean = 1.9%, SD = 6.4 of scat contents, Table 1).

The Gila River black bears exist on the periphery of black bear realized climatic niche space, in a hotter and drier climate than most bear occurrences (Figure 2, Table A1). This region of niche space has been rarely studied. We identified 46 primary studies on American black bear diets (see Table S1). While some studies occurred in higher elevation habitats within the Southwestern United States and Northwestern Mexico, nothing is known about the diet of black bears in climatic conditions similar to those experienced by the Gila River bears (Figure 2, Table S1).

## 4 DISCUSSION

**FIGURE 3:**
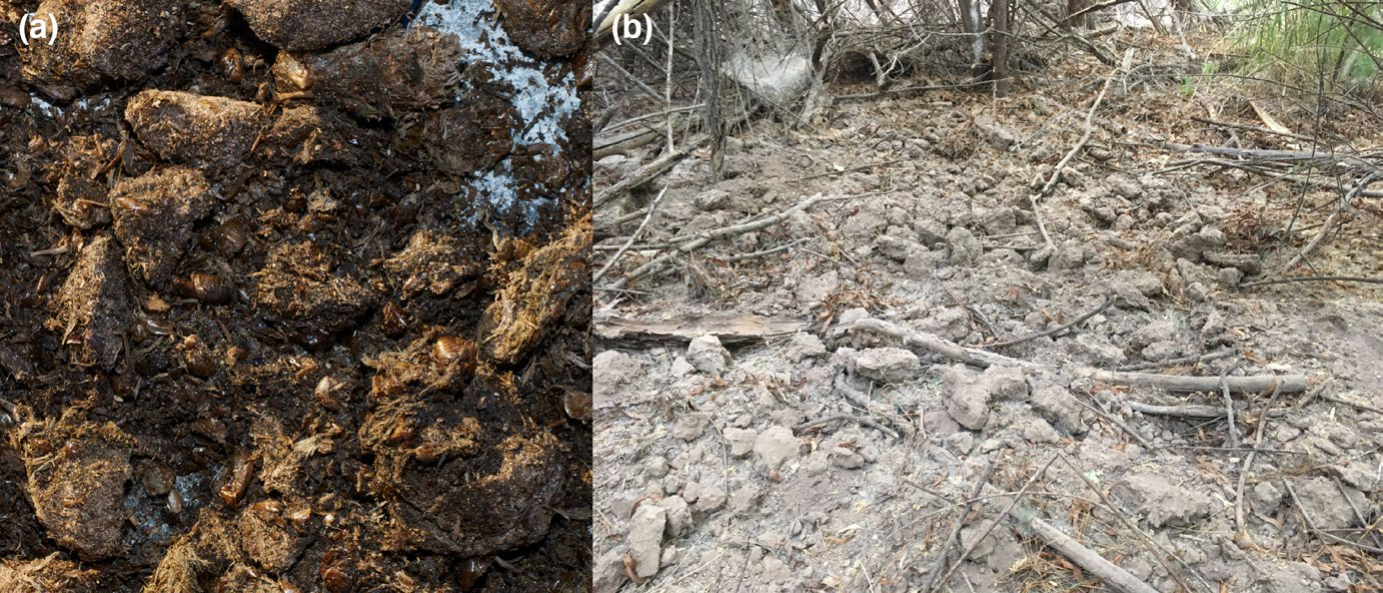
Cicadas as an important food item for black bears on a desert river. (a) Image of black bear scat, which was almost exclusively composed of cicada nymphs. (b) Extensive bioturbation of riverine soils, at times over areas >100 m^2^, may have important effects on nutrient availability, soil stability, vegetation, and the fluvial geomorphology of this system

The cicada‐dominated scat contents of the Gila River black bears are distinct (Figure 3a). While bear diet composition shows substantial variation (Costello et al., 2016; Ditmer et al., 2016), most reported American black bear diets are dominated not by arthropods but by energy‐rich fruits and seeds supplemented by herbaceous biomass (Fortin et al., 2013; Graber & White, 1983). While black bears do consume a variety of arthropods (Costello et al., 2016; Graber & White, 1983), in most studies arthropods constitute only a small proportion of each scat's contents (e.g., Baldwin & Bender, 2009; Bull & Torgersen, 2001; Graber & White, 1983; Greenleaf et al., 2009; Holcroft & Herrero, 1991; Juárez‐Casillas & Varas, 2013; Moeller et al., 2017). Arthropod consumption tends to increase during the summer months when there is surge in insect populations (reviewed in Graber and White (1983) and see Noyce et al. (1997), Coop et al. (2005), and Auger et al. (2004)). Some bear populations have also been documented consuming honeybees (Graber & White, 1983), potentially as bycatch during honey raids. The presence of honeybee exoskeletons in one third of the Gila River bear scats suggests that these bears may also be predating honeybee colonies.

The previously documented diets of bears in the Southwestern United States and Northwestern Mexico show bears primarily consuming energy‐rich nuts and seeds of oak (*Quercus* spp.), juniper (*Juniperus* spp.), and madrone (*Arbutus arizonica*) (López‐González et al., 2009; Sierra‐Corona et al., 2005). Indeed, reproductive success of black bears in New Mexico has been linked to acorn and juniper berry mast abundance (Costello et al., 2001). At lower elevations (ca. 2000 m.) in this region, black bear diets have been described as dominated by mesquite pods (*Prosopis* spp.), sotol (*Dasylirion* spp.), and *Yucca* spp. (Onorato et al., 2003). While a single black bear was observed consuming army cutworm moths (*Euxoa auxiliaris*) in subalpine habitat in New Mexico (Coop et al., 2005) and ant pupa have been recorded in 33% of bear scats in forested habitats in Utah (Auger et al., 2004), to the best of our knowledge, arthropods have not been documented as a comparably dominant dietary item for bears in this region.

Significant cicada nymph consumption has been recorded for brown bears (*Ursus arctos*) in mesic environments in Eurasia (Tomita & Hiura, 2020). Yet, while cicadas have previously been recorded in black bear scats in northern Mexican forests and chaparral, they were of minor dietary importance and these studies did not report whether these were adults or nymphs (Rodríguez‐Martínez et al., 2008; Sierra‐Corona et al., 2005). Some bear populations rely on nutrient‐rich and abundant arthropod food sources, such as the annual migration of grizzly bears to consume high‐elevation cutworm moths in the Yellowstone ecosystem (French et al., 1994). Cicada nymphs could be a similar resource for black bears within the Gila River region, leading to seasonal movements from higher elevation habitats (montane woodland is ~10.25 km from Gila River, Figure 1) or perhaps even freeing them from their typical habitat affinities. Apache cicada cohorts emerge after 3–4 years underground (Sanborn & Phillips, 2013). Nymphs are thus available subsurface year‐round, suggesting that they could enable year‐round bear occupancy. Although riparian habitats within the desert matrix were previously thought to be movement corridors (Costello et al., 2001), our data show they also support abundant insect prey for black bears.

Cicadas are considered ecologically important species as they connect groundwater to surface soil layers through the exudation of excess water and sugar as nymphs (Andersen, 1994). Likewise, adult Apache cicadas also provide significant food pulses for a number of above‐ground consumers, including USFWS‐listed Yellow‐billed Cuckoos (*Coccyzus americanus*) and Mississippi Kites (*Ictinia mississippiensis*) during their annual emergence (Glinski & Ohmart, 1984; Rosenberg et al., 1982). In addition to these species, we suggest that cicadas may facilitate the presence of black bears in this atypical habitat.

The excavation of cicadas produced large zones of overturned soil, reminiscent of wild boar rooting disturbances (Figure 3b). These zones were common along floodplain terraces, in both mesic cottonwood stands and on higher elevation tamarisk‐dominated (*Tamarix* spp.) terraces. This form of bioturbation may influence nutrient cycling through soil and litter mixing and could influence geomorphic responses to floods (Gabet et al., 2003; Lacki & Lancia, 1986; Naiman & Rogers, 1997). In fact, the germination of many foundational riparian tree species of this region, notably Fremont cottonwood and Goodding's willow, require bare moist substrate, generally provided by scouring floods (Shafroth et al., 2017) or at times by animal disturbance (Lundgren et al., 2021). By exposing bare substrate, bear bioturbation could potentially facilitate the germination of these ecologically important trees following more moderate flood events, which may otherwise be insufficient to remove leaf litter and competing vegetation (González et al., 2018; Shafroth et al., 2017; Stromberg et al., 1991).

Black bears are considered a population of conservation concern in the Southwestern United States and endangered in Northern Mexico (Monroy‐Vilchis et al., 2016), where they continue to be persecuted to protect livestock (Bravo & Davis, 2017; Varas, 2007). Black bears also face threats from landscape fragmentation, militarization of the United States–Mexico border, and potentially from poaching for gall bladders used in Eastern Traditional Medicine (Delfín‐Alfonso et al., 2012; Espinoza et al., 1993; McCracken et al., 1995; Onorato et al., 2004; Varas, 2007). The majority of discussion regarding the conservation of black bears in Southwestern North America has focused on the preservation of mountain island habitats and connectivity between them (Atwood et al., 2011; Delfín‐Alfonso et al., 2012; Monroy‐Vilchis et al., 2016). Our results suggest that desert rivers may be more important for black bears than often considered.

Our concepts of the habitat affinities and constraints of species can be influenced by historic and prehistoric anthropogenic legacies (Silliman et al., 2018) and by sampling biases (Britnell et al., 2021). This can stymie empirical understandings of ecological change, even leading to efforts to eradicate species perceived as non‐native in seemingly novel habitats (e.g., List et al., 2007; Martin et al., 2017). Given our small sample size, additional research is necessary to better understand black bears at the margins of their environmental distribution. Regardless, our results suggest the possibility that riparian habitats, in even the hottest North American deserts, may have once been and may continue to be habitat for American black bears.

## CONFLICT OF INTEREST

The authors declare no conflict of interest.

## AUTHOR CONTRIBUTIONS

**Erick J. Lundgren:** Conceptualization (equal); writing—original draft (equal); writing—review and editing (equal); methodology (equal); data curation (equal); formal analysis (lead); visualization (lead). **Karla T. Moeller:** Conceptualization (equal); methodology (equal); writing—original draft (equal); data curation (equal); writing—review and editing (equal). **Michael Otis Clyne:** Methodology (equal); data curation (equal); writing—review and editing (equal). **Owen S. Middleton:** Methodology (equal); data curation (equal); writing—review and editing (equal). **Sean M. Mahoney:** Conceptualization (equal); writing—review and editing (equal). **Christina L. Kwapich:** Conceptualization (equal); methodology (equal); writing—original draft (equal); writing—review and editing (equal); supervision (lead).

## Supporting information

Table S1

## Data Availability

All data are available on Figshare (https://doi.org/10.6084/m9.figshare.17392547).

## APPENDIX A

**TABLE A1:** Factor loadings of the Principle Components (PC1‐4) for the bioclimatic variables across black bear occurrence records (GBIF) and published dietary studies (Table S1)

Bioclimatic variable	PC1	PC2	PC3	PC4
Annual °C (bio1)	−0.275	−0.218	0.21	0.027
Diurnal °C range (bio2)	0.033	−0.357	0.041	−0.137
Isothermality (bio3)	−0.229	−0.281	−0.118	−0.093
°C seasonality (bio4)	0.315	0.077	0.152	0.196
Max °C warmest month (bio5)	−0.111	−0.315	0.293	0.051
Min °C coldest month (bio6)	−0.333	−0.138	0.026	−0.074
Temp. annual range (bio7)	0.318	−0.038	0.153	0.118
°C wettest quarter (bio8)	0.016	−0.159	0.37	0.499
°C driest quarter (bio9)	−0.288	−0.144	−0.118	−0.244
°C warmest quarter (bio10)	−0.152	−0.237	0.361	0.146
°C coldest quarter (bio11)	−0.319	−0.184	0.064	−0.069
Annual precipitation (bio12)	−0.262	0.282	0.026	0.132
Precipitation wettest month (bio13)	−0.271	0.212	−0.13	0.311
Precipitation driest month (bio14)	−0.139	0.295	0.308	−0.226
Precipitation seasonality (bio15)	−0.05	−0.218	−0.35	0.489
Precipitation wettest quarter (bio16)	−0.271	0.213	−0.133	0.305
Precipitation driest quarter (bio17)	−0.154	0.3	0.294	−0.201
Precipitation warmest quarter (bio18)	−0.088	0.224	0.385	0.164
Precipitation coldest quarter (bio19)	−0.266	0.199	−0.186	0.103
Standard deviation	2.793	2.279	1.802	1.046
Eigenvalues	7.80	5.19	3.25	1.09
Variation explained (%)	41.0	27.3	17.1	5.8
Cumulative variation explained)	41.0	68.4	85.5	91.2

Note

Axis standard deviation, eigenvalues, variation explained, and cumulative variation explained are at the bottom of the table.
