# Supplementary material for: Cicada nymphs dominate American black bear diet in a desert riparian area
Source: Ecol Evol. 2022 Mar 1;12(3):e8577. doi: 10.1002/ece3.8577 (PMC8888249; doi:10.1002/ece3.8577)
Supplement: Supplementary file 1 — Table S1 [file ECE3-12-e8577-s001.docx]

Supplementary information for

**Cicada nymphs dominate American black bear diet in a desert riparian area**

Erick J. Lundgren^*1,2,^ Karla T. Moeller^3^, Michael Otis Clyne^4^, Owen S. Middleton^5^, Sean M. Mahoney^6^, Christina L. Kwapich^7^

**Table S1.** References of American black bear diet and foraging studies across their distribution, with longitude and latitude. Note that studies are repeated for separate study areas.

| **Source** | **Longitude** | **Latitude** |  |
| --- | --- | --- | --- |
| Reimchen, T. E. "Nocturnal foraging behaviour of Black Bears, *Ursus americanus*, on Moresby Island, British Columbia." Canadian Field-Naturalist 112.3 (1998): 446-450. | -131.35 | 52.3666667 |  |
| Faison, Edward K., and David R. Houston. "Black bear foraging in response to beech bark disease in northern Vermont." Northeastern Naturalist 11.4 (2004): 387-394. | | -72.583333 | 45.9166667 |
| Smith, Douglas W., et al. "Black bear predation on beavers on an island in Lake Superior." American Midland Naturalist (1994): 248-255. | | -90.575139 | 46.9377778 |
| Harestad, Alton S., and Helen Davis. "Cannibalism by black bears in the Nimpkish Valley, British Columbia." (1996). | | -126.94414 | 50.2946111 |
| Fortin, Jennifer K., et al. "Dietary adjustability of grizzly bears and American black bears in Yellowstone National Park." The Journal of wildlife management 77.2 (2013): 270-281. | | -110.55544 | 44.2424167 |
| Popp, J. N., et al. "Black bear (*Ursus americanus*) and wolf (*Canis spp*.) summer diet composition and ungulate prey selectivity in Ontario, Canada." Mammal Research 63.4 (2018): 433-441. | | -81.687667 | 48.4850833 |
| Connor, Joseph P., et al. "Anthropogenic Influence on American Black Bear Diet in the Western Ozark Mountains in Eastern Oklahoma." Proceedings of the Oklahoma Academy of Science. Vol. 98. 2019. | | -93.546389 | 35.9467778 |
| Merkle, Jerod A., et al. "Dietary niche partitioning among black bears, grizzly bears, and wolves in a multiprey ecosystem." Canadian Journal of Zoology 95.9 (2017): 663-671. | | -133.23144 | 59.5713333 |
| Costello, Cecily M., et al. "Diet and macronutrient optimization in wild ursids: a comparison of grizzly bears with sympatric and allopatric black bears." PLoS one 11.5 (2016): e0153702. | | -110.97761 | 43.5371389 |
| Ditmer, Mark A., et al. "Are American black bears in an agricultural landscape being sustained by crops?." Journal of Mammalogy 97.1 (2016): 54-67. | | -96.3685 | 48.5289722 |
| Hopkins III, John B., and Carolyn M. Kurle. "Measuring the realized niches of animals using stable isotopes: from rats to bears." Methods in Ecology and Evolution 7.2 (2016): 210-221. | | -119.30428 | 37.8787222 |
| Lafferty, Diana JR, et al. "Sex, diet, and the social environment: factors influencing hair cortisol concentration in free-ranging black bears (Ursus americanus)." PLoS one 10.11 (2015): e0141489. | | -122.6 | 54.65 |
| Lesmerises, R√©mi, et al. "Linking GPS telemetry surveys and scat analyses helps explain variability in black bear foraging strategies." PLoS One 10.7 (2015): e0129857. | | -70.05 | 48.7 |
| Teunissen van Manen, Jennapher L., et al. "Using stable isotopes to assess dietary changes of American black bears from 1980 to 2001." Isotopes in environmental and health studies 50.3 (2014): 382-398. | | -83.720222 | 35.6173611 |
| Ju√°rez-Casillas, Luis Antonio, and Cora Varas. "Evaluation of black bear (Ursus americanus) diet and consequences in its conservation in Sierra de Picachos, Nuevo Leon, Mexico." Revista mexicana de biodiversidad 84.3 (2013): 970-976. | | -99.715833 | 25.8486111 |
| Seger, R. L., et al. "Body mass and mast abundance influence foraging ecology of the American black bear (Ursus americanus) in Maine." Canadian Journal of Zoology 91.7 (2013): 512-522. | | -68.744861 | 46.6040278 |
| Seger, R. L., et al. "Body mass and mast abundance influence foraging ecology of the American black bear (Ursus americanus) in Maine." Canadian Journal of Zoology 91.7 (2013): 512-522. | | -67.960528 | 44.9647778 |
| Seger, R. L., et al. "Body mass and mast abundance influence foraging ecology of the American black bear (Ursus americanus) in Maine." Canadian Journal of Zoology 91.7 (2013): 512-522. | | -68.908083 | 45.0773889 |
| Romain, Derrick A., Martyn E. Obbard, and James L. Atkinson. "Temporal variation in food habits of the American black bear (Ursus americanus) in the boreal forest of northern Ontario." The Canadian Field-Naturalist 127.2 (2013): 118-130. | | -83.333333 | 48.1666667 |
| Hopkins III, John B., et al. "Stable isotopes to detect food-conditioned bears and to evaluate human-bear management." The Journal of wildlife management 76.4 (2012): 703-713. | | -119.37433 | 37.9364167 |
| McLellan, Bruce N. "Implications of a high-energy and low-protein diet on the body composition, fitness, and competitive abilities of black (Ursus americanus) and grizzly (Ursus arctos) bears." Canadian Journal of Zoology 89.6 (2011): 546-558. | | -114.51111 | 49.2586667 |
| McLellan, Bruce N. "Implications of a high-energy and low-protein diet on the body composition, fitness, and competitive abilities of black (Ursus americanus) and grizzly (Ursus arctos) bears." Canadian Journal of Zoology 89.6 (2011): 546-558. | | -114.51111 | 49.2586667 |
| McLellan, Bruce N. "Implications of a high-energy and low-protein diet on the body composition, fitness, and competitive abilities of black (Ursus americanus) and grizzly (Ursus arctos) bears." Canadian Journal of Zoology 89.6 (2011): 546-558. | | -117.02478 | 51.2630556 |
| Merkle, Jerod A., Jonathan J. Derbridge, and Paul R. Krausman. "Using stable isotope analysis to quantify anthropogenic foraging in black bears." Human-Wildlife Interactions 5.1 (2011): 159-167. | | -114.25867 | 46.9025833 |
| Hatch, K. A., et al. "Isotopic and gross fecal analysis of American black bear scats." Ursus 22.2 (2011): 133-140. | | -109.052 | 39.3633889 |
| Baldwin, Roger A., and Louis C. Bender. "Foods and nutritional components of diets of black bear in Rocky Mountain National Park, Colorado." Canadian Journal of Zoology 87.11 (2009): 1000-1008. | | -105.61511 | 40.3436111 |
| Greenleaf, Schuyler S., et al. "Food habits of American black bears as a metric for direct management of human-bear conflict in Yosemite Valley, Yosemite National Park, California." Ursus 20.2 (2009): 94-101. | | -119.25142 | 37.8768333 |
| Thiemann, Gregory W., et al. "Trans fatty acids provide evidence of anthropogenic feeding by black bears." Human-Wildlife Conflicts 2.2 (2008): 183-193. | | -105.17167 | 39.4855278 |
| Mosnier, Arnaud, Jean-Pierre Ouellet, and Réhaume Courtois. "Black bear adaptation to low productivity in the boreal forest." Ecoscience 15.4 (2008): 485-497. | | -65.5 | 48.7666667 |
| Fortin, Jennifer K., et al. "Dietary and spatial overlap between sympatric ursids relative to salmon use." Ursus 18.1 (2007): 19-29. | | -150.64603 | 60.0230833 |
| Belant, Jerrold L., et al. "Interspecific resource partitioning in sympatric ursids." Ecological applications 16.6 (2006): 2333-2343. | | -150.50233 | 62.6361944 |
| Benson, John F., and Michael J. Chamberlain. "Food habits of Louisiana black bears (*Ursus americanus luteolus*) in two subpopulations of the Tensas River Basin." The American midland naturalist 156.1 (2006): 118-127. | | -91.359417 | 32.2370278 |
| Ziegltrum, Georg J. "Efficacy of black bear supplemental feeding to reduce conifer damage in western Washington." The Journal of wildlife management 68.3 (2004): 470-474. | | -123.75269 | 48.1281944 |
| Ziegltrum, Georg J. "Efficacy of black bear supplemental feeding to reduce conifer damage in western Washington." The Journal of wildlife management 68.3 (2004): 470-474. | | -124.35267 | 47.6017222 |
| Iverson, Sara J., J. E. McDonald, Jr, and L. K. Smith. "Changes in the diet of free-ranging black bears in years of contrasting food availability revealed through milk fatty acids." Canadian Journal of Zoology 79.12 (2001): 2268-2279. | | -72.683333 | 42.45 |
| Partridge, Steven T., et al. "Impacts of supplemental feeding on the nutritional ecology of black bears." (2001). | | -123.10081 | 46.8681667 |
| Stratman, Mary R., and Michael R. Pelton. "Feeding ecology of black bears in northwest Florida." Florida Field Naturalist 27.3 (1999): 95-102. | | -86.550472 | 30.4596111 |
| Payne, Neil F., Bruce E. Kohn, and Ned C. Norton. "Black Bear Food Items in Northern Wisconsin." TRANSACTIONS 86 (1998): 263. | | -90.387611 | 46.3955 |
| Roof, Jadye C. Black bear food habits in the Lower Wekiva River Basin of central Florida. Florida Game and Fresh Water Fish Commission, 1997. | | -81.391056 | 28.8663056 |
| Kasbohm, John W., James G. Kraus, and Michael R. Vaughan. "Food habits and nutrition of black bears during a gypsy moth infestation." canadian Journal of Zoology 73.9 (1995): 1771-1775. | | -78.465028 | 38.4346944 |
| Stubblefield, Cynthia H. "Food habits of black bear in the San Gabriel Mountains of southern California." The Southwestern Naturalist 38.3 (1993): 290-293. | | -117.75 | 34.25 |
| Hellgren, Eric C. "Status, distribution, and summer food habits of black bears in Big Bend National Park." The Southwestern Naturalist 38.1 (1993): 77-80. | | -103.27861 | 29.2815278 |
| Hellgren, Eric C., Michael R. Vaughan, and Roy L. Kirkpatrick. "Seasonal patterns in physiology and nutrition of black bears in Great Dismal Swamp, Virginia–North Carolina." Canadian Journal of Zoology 67.8 (1989): 1837-1850. | | -76.441944 | 36.5616111 |
| Hellgren, Eric C., and Michael R. Vaughan. "Seasonal food habits of black bears in Great Dismal Swamp, Virginia–North Carolina." Proceedings of the Annual conference of the southeastern Association of Fish and Wildlife Agencies. Vol. 42. 1988. | | -76.441944 | 36.5616111 |
| MacHutchon, A. Grant. "Spring and summer food habits of black bears in the Pelly River Valley, Yukon." (1989). | | -133.5 | 62 |
| McDonald Jr, John E., and Todd K. Fuller. "Effects of spring acorn availability on black bear diet, milk composition, and cub survival." Journal of Mammalogy 86.5 (2005): 1022-1028. | | -72.683333 | 42.45 |
| Quinn, Thomas P., et al. "Transportation of Pacific salmon carcasses from streams to riparian forests by bears." Canadian Journal of Zoology 87.3 (2009): 195-203. | | -135.77786 | 57.8955833 |
| Fox, Caroline Hazel, Paul Charles Paquet, and Thomas Edward Reimchen. "Novel species interactions: American black bears respond to Pacific herring spawn." BMC ecology 15.1 (2015): 14. | | -127.60483 | 50.5193056 |
| Klinka, D. R., and T. E. Reimchen. "Darkness, twilight, and daylight foraging success of bears (Ursus americanus) on salmon in coastal British Columbia." Journal of Mammalogy 90.1 (2009): 144-149. | | -128.97111 | 53.4238889 |
| Grinath, Joshua B., Brian D. Inouye, and Nora Underwood. "Bears benefit plants via a cascade with both antagonistic and mutualistic interactions." Ecology Letters 18.2 (2015): 164-173. | | -106.816 | 38.719 |
| Mattson, David J., et al. "Bear feeding activity at alpine insect aggregation sites in the Yellowstone ecosystem." Canadian Journal of Zoology 69.9 (1991): 2430-2435. | | -109.33781 | 43.9684444 |
| Moeller, Karla T., et al. "Observation of an American black bear eating odonates in Yosemite National Park." Western North American Naturalist 77.1 (2017): 99-101. | | -119.34039 | 37.8900278 |
| Jones, Robert W., et al. "Black bears feed on harvestmen (Opiliones) in northwestern Mexico." The Journal of Arachnology 44.1 (2016): 83-84. | | -108.82 | 31.12 |
| López-González, Carlos A., et al. "Scorpions are a food item of American black bears in Sonora, Mexico." Western North American Naturalist 69.1 (2009): 131-133. | | -108.91667 | 31.1666667 |
| Auger, Janene, et al. "Selection of ants by the American black bear (Ursus americanus)." Western North American Naturalist (2004): 166-174. | | -109 | 38.25 |
| Noyce, Karen V., Paul B. Kannowski, and Michael R. Riggs. "Black bears as ant-eaters: seasonal associations between bear myrmecophagy and ant ecology in north-central Minnesota." Canadian Journal of Zoology 75.10 (1997): 1671-1686. | | -93.5 | 47.5 |
| Coop, Jonathan D., et al. "Black bears forage on army cutworm moth aggregations in the Jemez Mountains, New Mexico." The Southwestern Naturalist 50.2 (2005): 278-281. | | -106.40333 | 35.8894722 |
| Doan-Crider, Diana L., Andrew N. Tri, and David G. Hewitt. "Woody cover and proximity to water increase American black bear depredation on cattle in Coahuila, Mexico." Ursus 28.2 (2017): 208-217. | | -101.78661 | 28.9529444 |
| van Manen, Jennapher Teunissen, et al. "Assessing longitudinal diet patterns of black bears in Great Smoky Mountains National Park using stable carbon and nitrogen isotopes." International Bear News: 37. | | -83.579389 | 35.5796389 |
| Boileau, F., M. Crête, and J. Huot. "Food habits of the black bear, Ursus americanus, and habitat use in Gaspesie Park, eastern Quebec." Canadian field-naturalist. Ottawa ON 108.2 (1994): 162-169. | | -65.827528 | 48.6598611 |
| Grenfell Jr, William E., and Allan J. Brody. "Seasonal foods of black bears in Tahoe National Forest, California." California Fish and Game 69.3 (1983): 132-150. | | -120.51619 | 39.4182778 |
| Warburton, G. S. "Contents of black bear scats from the central Adirondacks in late summer [New York]." New York Fish and Game Journal (1982). | | -74.680556 | 43.9086111 |
| Corona, Rodrigo Sierra, et al. "Black bear abundance, habitat use, and food habits in the Sierra San Luis, Sonora, Mexico." Connecting Mountain Islands and Desert Seas: Biodiversity and Management of the Madrean Archipelago II and 5th Conference on Research and Resource Management in the Southwestern Deserts: May 11-15, 2004, Tucson, Arizona. US Department of Agriculture, Forest Service, Rocky Mountain Research Station, 2005. | | -31.196944 | 108.946111 |
| Tozer, Douglas C., et al. "Managing ecological traps: logging and sapsucker nest predation by bears." The Journal of Wildlife Management 76.5 (2012): 887-898. | | -78.410361 | 45.8118056 |
| Tozer, Douglas C., et al. "Predation by bears on woodpecker nests: are nestling begging and habitat choice risky business?." The Auk 126.2 (2009): 300-309. | | -78.410361 | 45.8118056 |
| Kimball, Bruce A., et al. "Chemically mediated foraging preference of black bears (*Ursus americanus*)." Journal of Mammalogy 79.2 (1998): 448-456. | | -122.91667 | 46.1666667 |
| Kimball, Bruce A., et al. "Chemically mediated foraging preference of black bears (*Ursus americanus*)." Journal of Mammalogy 79.2 (1998): 448-456. | | -121.75 | 47.1666667 |
| Kimball, Bruce A., et al. "Chemically mediated foraging preference of black bears (*Ursus americanus*)." Journal of Mammalogy 79.2 (1998): 448-456. | | -122.08333 | 46.5 |
| Kimball, Bruce A., et al. "Chemically mediated foraging preference of black bears (*Ursus americanus*)." Journal of Mammalogy 79.2 (1998): 448-456. | | -121.66667 | 48.5 |
| Kimball, Bruce A., et al. "Chemically mediated foraging preference of black bears (*Ursus americanus*)." Journal of Mammalogy 79.2 (1998): 448-456. | | -122.66667 | 44.6666667 |
| Kimball, Bruce A., et al. "Chemically mediated foraging preference of black bears (*Ursus americanus*)." Journal of Mammalogy 79.2 (1998): 448-456. | | -122.5 | 44.8333333 |
| Kimball, Bruce A., et al. "Chemically mediated foraging preference of black bears (*Ursus americanus*)." Journal of Mammalogy 79.2 (1998): 448-456. | | -122.83333 | 47.8333333 |
| Kimball, Bruce A., et al. "Chemically mediated foraging preference of black bears (*Ursus americanus*)." Journal of Mammalogy 79.2 (1998): 448-456. | | -123.83333 | 44.75 |
| Kimball, Bruce A., et al. "Chemically mediated foraging preference of black bears (*Ursus americanus*)." Journal of Mammalogy 79.2 (1998): 448-456. | | -121.75 | 47.5833333 |
| Harrer, Laurie EF, and Taal Levi. "The primacy of bears as seed dispersers in salmon–bearing ecosystems." Ecosphere 9.1 (2018): e02076. | | -135.35564 | 59.1391667 |
| Lovich, Jeffrey E., et al. "Black bears (*Ursus americanus*) as a novel potential predator of Agassiz's desert tortoises (*Gopherus agassizii*) at a California wind energy facility." Bulletin, Southern California Academy of Sciences 113.1 (2014): 34-41. | | -116.66722 | 33.9516667 |
| Hamer, David, and Ian Pengelly. "Whitebark Pine (*Pinus albicaulis*) seeds as food for bears (*Ursus* spp.) in Banff National Park, Alberta." The Canadian Field-Naturalist 129.1 (2015): 8-14. | | -115.90739 | 51.4931111 |
| Mattson, David J., and Terence A. Arundel. "Consumption of seeds of southwestern white pine (*Pinus* *strobiformis*) by black bear (*Ursus americanus*)." The Southwestern Naturalist 58.2 (2013): 243-245. | | -111.67011 | 35.3613889 |
| Willson, Mary F., et al. "Black bear (*Ursus americanus*) foraging on Black cottonwood (*Populus* *trichocarpa*) catkins in Southeast Alaska." Northwestern Naturalist 93.3 (2012): 211-219. | | -134.54422 | 58.4176667 |
| Borchert, M. A. R. K., FRANK W. Davis, and Jason Kreitler. "Carnivore use of an avocado orchard in southern California." California Fish and Game 94.2 (2008): 61-74. | | -119.26636 | 34.3828611 |
| Kuhn, Kellie M., and Stephen B. Vander Wall. "Black bears (*Ursus* *americanus*) harvest Jeffrey pine (*Pinus jeffreyi*) seeds from tree canopies." Western North American Naturalist 67.3 (2007): 384-388. | | -119.87639 | 39.2527778 |
| Svoboda, Nathan J., et al. "American black bear predation of an adult white-tailed deer." Ursus 22.1 (2011): 91-94. | | -87.346389 | 45.5705556 |
| Garneau, Danielle E., et al. "Spatio-temporal patterns of predation among three sympatric predators in a single-prey system." Wildlife Biology 13.2 (2007): 186-194. | | -161.19019 | 61.5029444 |
| Walley, William J. "Probable black bear, *Ursus americanus*, retrieval of an elk, *Cervus elaphus*, carcass from a small lake in Riding Mountain National Park, Manitoba." The Canadian Field-Naturalist 120.1 (2006): 110-112. | | -99.926417 | 50.7169722 |
| Kunkel, Kyran E., and L. David Mech. "Wolf and bear predation on white-tailed deer fawns in northeastern Minnesota." Canadian Journal of Zoology 72.9 (1994): 1557-1565. | | -92 | 48 |
| Mathews, Nancy E., and William F. Porter. "Black bear predation of white-tailed deer neonates in the central Adirondacks." Canadian Journal of Zoology 66.5 (1988): 1241-1242. | | -74.622889 | 43.9006944 |
| Ozoga, John J., and Louis J. Verme. "Predation by black bears on newborn white-tailed deer." Journal of Mammalogy 63.4 (1982): 695-695. | | -85.98575 | 46.2450556 |
| Reimchen, T. E. "Nocturnal foraging behaviour of Black Bears, *Ursus americanus*, on Moresby Island, British Columbia." Canadian Field-Naturalist 112.3 (1998): 446-450. | | -131.35 | 52.3666667 |
| Faison, Edward K., and David R. Houston. "Black bear foraging in response to beech bark disease in northern Vermont." Northeastern Naturalist 11.4 (2004): 387-394. | | -72.583333 | 45.9166667 |
| Fortin, Jennifer K., et al. "Dietary adjustability of grizzly bears and American black bears in Yellowstone National Park." The Journal of wildlife management 77.2 (2013): 270-281. | | -110.28492 | 44.5603056 |
| Costello, Cecily M., et al. "Diet and macronutrient optimization in wild ursids: a comparison of grizzly bears with sympatric and allopatric black bears." PLoS one 11.5 (2016): e0153702. | | -110.35553 | 44.1590833 |
| Lafferty, Diana JR, et al. "Sex, diet, and the social environment: factors influencing hair cortisol concentration in free-ranging black bears (*Ursus americanus*)." PLoS one 10.11 (2015): e0141489. | | -122.6 | 54.65 |
| Lesmerises, R√©mi, et al. "Linking GPS telemetry surveys and scat analyses helps explain variability in black bear foraging strategies." PLoS One 10.7 (2015): e0129857. | | -70.7 | 49.2833333 |
| Ju√°rez-Casillas, Luis Antonio, and Cora Varas. "Evaluation of black bear (*Ursus americanus*) diet and consequences in its conservation in Sierra de Picachos, Nuevo Leon, Mexico." Revista mexicana de biodiversidad 84.3 (2013): 970-976. | | -100.11111 | 26.3227778 |
| Romain, Derrick A., Martyn E. Obbard, and James L. Atkinson. "Temporal variation in food habits of the American black bear (*Ursus americanus*) in the boreal forest of northern Ontario." The Canadian Field-Naturalist 127.2 (2013): 118-130. | | -83.333333 | 48.1666667 |
| Hatch, K. A., et al. "Isotopic and gross fecal analysis of American black bear scats." Ursus 22.2 (2011): 133-140. | | -109.52 | 39.5976111 |
| Mosnier, Arnaud, Jean-Pierre Ouellet, and Rehaume Courtois. "Black bear adaptation to low productivity in the boreal forest." *Ecoscience* 15.4 (2008): 485-497. | | -65.5 | 48.7666667 |
| Iverson, Sara J., J. E. McDonald, Jr, and L. K. Smith. "Changes in the diet of free-ranging black bears in years of contrasting food availability revealed through milk fatty acids." Canadian Journal of Zoology 79.12 (2001): 2268-2279. | | -72.683333 | 42.45 |
| Stubblefield, Cynthia H. "Food habits of black bear in the San Gabriel Mountains of southern California." The Southwestern Naturalist 38.3 (1993): 290-293. | | -117.75 | 34.25 |
| MacHutchon, A. Grant. "Spring and summer food habits of black bears in the Pelly River Valley, Yukon." (1989). | | -133.5 | 62 |
| McDonald Jr, John E., and Todd K. Fuller. "Effects of spring acorn availability on black bear diet, milk composition, and cub survival." Journal of Mammalogy 86.5 (2005): 1022-1028. | | -72.683333 | 42.45 |
| Auger, Janene, et al. "Selection of ants by the American black bear (Ursus americanus)Western North American Naturalist (2004): 166-174. | | -109.5 | 38.6666667 |
